# Analysis of polar urinary metabolites for metabolic phenotyping using supercritical fluid chromatography and mass spectrometry

**DOI:** 10.1016/j.chroma.2016.04.040

**Published:** 2016-06-03

**Authors:** Arundhuti Sen, Christopher Knappy, Matthew R. Lewis, Robert S. Plumb, Ian D. Wilson, Jeremy K. Nicholson, Norman W. Smith

**Affiliations:** a Analytical and Environmental Sciences Division, Faculty of Life Sciences & Medicine, Franklin-Wilkins Building, King’s College London, London SE1 9NH, United Kingdom; b Computational and Systems Medicine, Department of Surgery and Cancer, Faculty of Medicine, Sir Alexander Fleming Building, Imperial College London, South Kensington Campus, London SW7 2DD, United Kingdom; c MRC-NIHR National Phenome Centre, Division of Computational and Systems Medicine, Department of Surgery and Cancer, IRDB Building, Imperial College London, Hammersmith Hospital, London W12 0NN, United Kingdom; d Waters Corporation, Milford, MA, USA

**Keywords:** SFC, Method development, Polar, Stationary phase, Modifier, Additive

## Abstract

Supercritical fluid chromatography (SFC) is frequently used for the analysis and separation of non-polar metabolites, but remains relatively underutilised for the study of polar molecules, even those which pose difficulties with established reversed-phase (RP) or hydrophilic interaction liquid chromatographic (HILIC) methodologies. Here, we present a fast SFC-MS method for the analysis of medium and high-polarity (−7 ≤ cLogP ≤ 2) compounds, designed for implementation in a high-throughput metabonomics setting. Sixty polar analytes were first screened to identify those most suitable for inclusion in chromatographic test mixtures; then, a multi-dimensional method development study was conducted to determine the optimal choice of stationary phase, modifier additive and temperature for the separation of such analytes using SFC. The test mixtures were separated on a total of twelve different column chemistries at three different temperatures, using CO_2_-methanol-based mobile phases containing a variety of polar additives. Chromatographic performance was evaluated with a particular emphasis on peak capacity, overall resolution, peak distribution and repeatability. The results suggest that a new generation of stationary phases, specifically designed for improved robustness in mixed CO_2_-methanol mobile phases, can improve peak shape, peak capacity and resolution for all classes of polar analytes. A significant enhancement in chromatographic performance was observed for these urinary metabolites on the majority of the stationary phases when polar additives such as ammonium salts (formate, acetate and hydroxide) were included in the organic modifier, and the use of water or alkylamine additives was found to be beneficial for specific subsets of polar analytes. The utility of these findings was confirmed by the separation of a mixture of polar metabolites in human urine using an optimised 7 min gradient SFC method, where the use of the recommended column and co-solvent combination resulted in a significant improvement in chromatographic performance.

## Highlights

• An SFC method has been developed for the analysis of polar urinary metabolites.
• 12 stationary phases, 9 modifier additives and 3 temperatures were evaluated.
• DIOL and 2-PIC columns provide highest peak capacity and overall resolution.
• Ammonium formate, ammonium hydroxide and water considerably improved separation.
• Alkylamine additives should be strongly considered for polar SFC-UV analysis.

## 1 Introduction

Supercritical fluid chromatography (SFC) as a viable separation technique was first reported by Klesper et al. in 1962 [1]; the decades since have seen a steady increase in its reported use [2] for an ever-expanding range of applications [3]. However, unlike liquid chromatography (LC) or gas chromatography (GC), SFC has remained a niche technique for most of its history, primarily used for chiral separations or for preparative chromatography in industrial settings. Instrumental limitations played an important role in slowing the growth of analytical SFC, as many early instruments exhibited (amongst other issues) poor pump performance resulting in unstable backpressures [4]. An important factor driving the current resurgence of interest in the field has thus been the development of a new generation of SFC instruments, which provide substantial improvements in system reliability and performance over their predecessors, and are supplied with purpose-built interfaces for facile coupling to mass spectrometers [5]. These new instruments have been developed at a time of significant advances in column technology, including the widespread availability of columns packed with sub–3 μm porous shell particles and sub–2 μm totally porous particles [6]. Column manufacturers have responded to the surge in SFC use by offering columns specifically designed for improved stability in mixed CO_2_-methanol mobile phases [7]. Considered together, these developments indicate that SFC has the potential to develop into a mainstream mode of chromatography and, as discussed in a recent comprehensive review of the technique, may yet become as valued and widespread a tool as reversed-phase (RP) LC [8].

SFC is often described as an alternative to normal phase chromatography, without the requirement for problematic organic solvents, such as heptane [9]. Several recently reported achiral SFC applications have involved relatively non-polar analytes (e.g. various classes of lipids [10], [11], [12], organic pesticides [13], fat-soluble vitamins [14] etc.). Polar analytes have been more sparingly studied, even though Berger and others have shown that moderately polar pharmaceutical compounds can be separated on polar stationary phases by the addition of organic co-solvents (‘modifiers’) containing selected additives to the CO_2_ mobile phase [15]. Modifier addition can increase the solubility of polar analytes in the mobile phase, and via adsorptive processes can alter stationary phase characteristics with consequent changes in retention, selectivity and efficiency [9], [16]. Methanol is a popular choice as mobile-phase modifier in SFC, as it has been shown to improve the efficiency and peak shape for polar analytes on a variety of stationary phases; this improvement has been attributed to the superior hydrogen-bonding ability of methanol in comparison to other tested modifiers such as acetonitrile [17]. Modifier enrichment with neutral, acidic or basic additives has also been shown to reduce peak tailing and retention for polar analytes to different extents, depending upon the additive type as well as analyte characteristics, including their octanol/water partition coefficients, relative acidity, molecular size and functional group topology [18], [19], [20]. These findings have led to the suggestion that, with the appropriate combination of stationary phase, modifier and modifier additive, SFC methods can be developed to replace many RPLC or hydrophilic interaction liquid chromatography (HILIC) methods for polar compound analysis [8].

One potential application area for SFC is metabolic phenotyping, also commonly referred to as metabonomics or metabolomics. Metabolic phenotyping determines metabolite profiles for biofluids and tissues in order to detect systemic responses to pathophysiological stimuli and to derive a comprehensive, systems-level understanding of health and disease [21]. Both targeted and untargeted metabolic phenotyping are beginning to be applied to very large-scale studies (ranging from many hundreds to thousands of samples), derived from preclinical, clinical and epidemiological investigations. Currently, the major analytical platforms used in metabolic phenotyping include nuclear magnetic resonance (NMR) spectroscopy, as well as GC and UHPLC coupled with mass spectrometry (i.e. GC–MS, UHPLC–MS) [22]. For UHPLC–MS-based profiling of polar, hydrophilic metabolites, HILIC and ion-pair chromatography (IPC) have been employed with some success. However, the analysis of hydrophilic metabolites remains challenging, since HILIC is not a solution for all compounds, and IPC can lead to problematic long-term contamination of the MS [23]. This represents a major difficulty for metabolic phenotyping, as many of these chromatographically challenging metabolites represent key intermediates within important anabolic and catabolic biological processes, such as central carbon metabolism. At present, the lack of readily implementable LC-based technologies for the comprehensive and routine profiling of these compounds is therefore a significant constraint on our ability to monitor some basic biochemical differences between normal and diseased (e.g. cancerous) cells. New separation strategies such as SFC, which increase metabolome coverage while reducing solvent requirements, are consequently of great interest to metabolic phenotyping studies.

Here, we report the development of an SFC-MS method for the analysis of polar analytes in urine, designed for implementation in a high-throughput metabolic phenotyping environment. An initial compound screening study, to identify polar analytes suitable for use in chromatographic test mixtures, was followed by a comprehensive method development study, designed to identify the best choice of stationary phase, modifier additive and temperature for the separation of test compounds using SFC. A total of twelve columns, nine modifier additives and three temperatures were tested during method development; methanol was used as the organic modifier throughout the study, with orthogonality and selectivity considerations driving the selection of both column and modifier additive. The chromatographic performance of each evaluated set of conditions (column, modifier and temperature) was judged based on calculated peak base widths, total resolution and the normalised product of the resolution, with the first two parameters used to compare relative peak capacities across conditions. The results suggest that a new generation of bridged ethylene hybrid (BEH) stationary phases, specifically designed for SFC analysis, is particularly well suited for the separation of a wide range of polar analytes, with diol and 2-picolylamine chemistries yielding significant improvements in chromatographic performance. It was also observed that the presence of ammonium formate, water or ammonium hydroxide in the methanol modifier can substantially improve chromatographic separation of highly polar metabolites. In addition, the use of alkylamines as modifier additives is strongly recommended for the analysis of basic analytes when the SFC is coupled to a UV–vis detector. The utility of these findings was confirmed by separating a subset of polar analytes in human urine using the Torus Diol column with 20 mM ammonium formate in methanol as an organic modifier, and comparing the results to conditions currently in general use for polar analyte separation on SFC.

## 2 Materials and methods

### 2.1 Solvents and solvent additives

LC–MS (Chromasolv) grade methanol, acetonitrile and isopropanol were purchased from Sigma-Aldrich (Poole, UK); water was obtained from an ultra-pure water purification system (Millipore, UK) and ‘food fresh’ liquid CO_2_ (99.8% purity) was obtained from BOC (UK). Formic acid (98%), acetic acid (for LC–MS), ammonium formate (≥99.0%), ammonium acetate (≥99.0%), ammonium hydroxide solution (∼10% in water, for HPLC), isopropylamine (99%), isobutylamine (99%) and isopentylamine (99%) were also purchased from Sigma.

### 2.2 Compound library screening

#### 2.2.1 Composition of screened library

Table S1 lists all sixty polar compounds tested during the compound screening study; these were obtained as pure solids of ≥95% purity from Sigma, with the exception of l-histidine and cytosine, which were purchased from Alfa Aesar (Heysham, UK). The selected compounds covered a cLogP range (calculated using ChemAxon [24]) from +1.2 to −6.5, a cLogD range (calculated using a pH of 5.5 and the ACD/Labs Percepta platform [25]) from 0.3 to −6.2, and molecular weights up to 505 Da 5 mL stock solutions of each of the 60 analytes were prepared in water or methanol and stored at −20 °C before use. Immediately prior to analysis, the stock solutions were diluted in 1:1 methanol:water to obtain a 10 μg/mL solution of each analyte; 1 μL of this dilute solution was injected onto the column for analysis.

#### 2.2.2 Chromatography for library screening

Compound screening was performed on a Waters ACQUITY UPC^2^, consisting of a binary solvent manager, a sample manager held at 4 °C and fitted with a 10 μL injection loop, an insulated column compartment with an active pre-heater and column heater, a PDA detector fitted with an 8 μL flow-cell, and a convergence manager containing an automated backpressure regulator (ABPR) with both static and dynamic components to control post-column system pressure. All injections were performed in partial loop (needle overfill) mode, and PDA data were collected between 190 and 400 nm for all analyses.

Compounds were analysed on a fully-porous 3.00 × 100 mm, 1.7 μm ACQUITY UPC^2^ BEH 2-ethylpyridine (BEH 2-EP) column maintained at 35 °C, using a mobile phase composed of CO_2_ (solvent A) and the selected organic modifier (solvent B) with the following 14.65 min gradient elution method: the mobile phase composition changed from 98% A at 0 min to 50% B at 10 min, was held at 50% B for 1 min, then returned to 98% A in 0.65 min, followed by re-equilibration at initial conditions till the end of the run. The flow-rate was 1 mL/min or 1.2 mL/min, depending upon whether isopropanol or methanol (respectively) was used as modifier. Four different organic modifiers were used to analyse each compound, *viz.* 5% v/v water in methanol, 20 mM ammonium formate in methanol, 0.2% v/v formic acid in methanol and 7% v/v water in isopropanol. Each of these modifiers and additives has been successfully employed elsewhere for SFC analysis of polar moieties [26], [15].

#### 2.2.3 Mass spectrometry for library screening

The Waters UPC^2^ was coupled to a Waters Quattro Premier tandem mass spectrometer via a purpose-built, two-part stainless steel splitter supplied by Waters. The flow from the PDA detector was mixed with 0.2 mL/min makeup solvent (0.1% v/v formic acid in methanol), supplied by a Waters 515 HPLC pump, and the ABPR was used to maintain the resultant solvent stream at a pressure of 2000 psi (138 bar) throughout the run. The pressurised stream was directed into the ESI sample capillary via a length of 50 μm PEEKsil tubing.

Compounds were detected by selected ion recording (SIR) in either positive or negative ESI MS mode, based on the known ionisation preference or optimal ionisation mode for each analyte. Source conditions in positive mode were as follows: capillary voltage 3.5 kV, cone voltage 30 V, source temperature 120 °C, desolvation temperature 250 °C, cone gas flow 300 L/hr and desolvation gas flow 700 L/hr. Source conditions for negative mode were as follows: capillary voltage 3.0 kV, cone voltage 30 V, source temperature 100 °C, desolvation temperature 250 °C, cone gas flow 50 L/hr, desolvation gas flow 500 L/hr. Analyser collision cell entrance and exit voltages were set at 50 V, with the collision cell voltage set at 2 V. SIR data was collected with a dwell time of 0.05 s and a cone voltage of 30 V (this was found to be suitable for the majority of analytes studied). Data acquisition, data handling and instrument operation were controlled by MassLynx (v. 4.1 from Waters, Milford MA, USA); raw SFC-MS and SFC-PDA data was subsequently processed and analysed using TargetLynx, a MassLynx module.

### 2.3 Method development

**Scheme 1:**
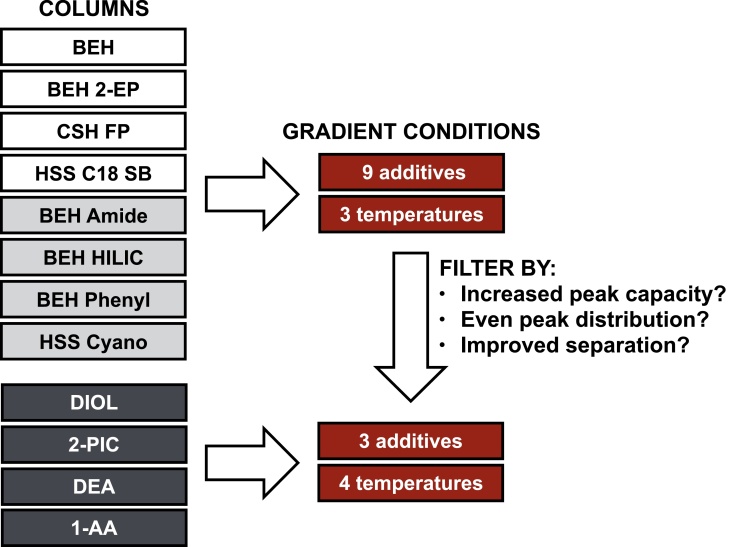
Experimental design used for chromatographic method development.

Scheme 1 illustrates the experimental design followed during chromatographic method development. An initial set of eight columns were screened in nine co-solvent additives and at three temperatures, with results evaluated based on improvements in peak capacity and peak distribution (*cf*. Section 3.2). The four Torus columns were released in October 2014, after the study had commenced; in order to minimise the total number of experiments, these columns were accordingly only screened in the three MS-compatible modifiers that had yielded the best performance till that date. In addition, changing the temperature in the range initially selected for evaluation (35–45 °C) did not lead to significant alterations in peak width or distribution. Consequently, the Torus columns were evaluated at one additional temperature, 55 °C, in the three selected co-solvents. Thus a total of 264 unique combinations of column, modifier additive and column temperature were tested as part of the method development study.

#### 2.3.1 Test mixture preparation and extraction of human urine

In the initial compound screening study, eleven compounds were identified as ‘responders’, which eluted in all four organic modifiers with acceptable *k* values and peak symmetries. An additional nine compounds eluted in all four modifiers with either a *k* or a b/a value that fell outside the acceptable limits for a ‘hit’; these were considered to be ‘sub-responders’. The eleven responders and nine sub-responders (a total of twenty compounds) were subsequently incorporated into five different test mixtures in 1:1 methanol:water, as described in Table S2.The test mixtures were designed to cover a range of cLogP values and compound classes, and all contained caffeine as an internal standard for the measurement of analytical repeatability. Test mixtures were prepared in bulk as 25 mL solutions, and stored in 1 mL aliquots at −20 °C until immediately before analysis.

For the separation of analytes in human urine, urine samples were obtained from healthy individuals and pooled to form a reference urine sample; this was spiked with caffeine, uridine and cytosine as aqueous stock solutions. The spiked urine was then mixed with methanol in a 1:3 urine:methanol ratio, vortexed for 30 s at room temperature, and centrifuged at 700*g* at 4 °C for 10 min. The centrifuged sample was divided into 1 mL aliquots and stored at −80 °C, then thawed immediately prior to analysis.

#### 2.3.2 Stationary phases

All columns screened during method development had dimensions of 3.00 mm i.d. × 100 mm, and were obtained from Waters (Milford, USA). In all, twelve fully-porous columns were tested, including four Acquity UPC^2^ columns (BEH 2-EP, BEH, CSH FP and HSS C_18_ SB), four Acquity UPLC columns (BEH Amide, BEH HILIC, BEH Phenyl and HSS Cyano), and four Acquity UPC^2^ Torus columns (2-picolylamine or 2-PIC, Diol, Diethylamine or DEA and 1-amino anthracene or 1-AA). Of these, only the HSS columns had a particle size (d_p_) of 1.8 μm, the remainder having a d_p_ of 1.7 μm.

The columns were tested for compliance with manufacturer QC specifications, and equilibrated in 100% CO_2_ at 1 mL/min for 60 min before initial use. Column performance was monitored at the start of each day of analysis using repeated injections of caffeine, adenosine and toluene (used as an approximate measure of column dead-volume by UV [27]) and an isocratic method with 5% (v/v) water in methanol as the organic modifier. Between analyses, columns were washed with 1:1 CO_2_:methanol at 1 mL/min for 50 min, then stored in 100% CO_2_.

#### 2.3.3 Modifier preparation

Methanol was used as the organic modifier throughout the method development phase, and contained one of nine different additives at a concentration of 0.5% v/v (for formic acid, acetic acid, isopropylamine, isobutylamine and isopentylamine), 20 mM (for ammonium formate, acetate and hydroxide), or 5% v/v (for water). All modifier solutions were prepared volumetrically or, in the case of the ammonium salts, by addition of the solid salt to the methanol, followed by sonication at room temperature for 10 min.

#### 2.3.4 Chromatography for method development

A 7.35 min gradient elution method was used for all method development analyses, *viz.* 98% CO_2_ at 0 min to 50% CO_2_ at 4 min, held at 50% CO_2_ for 1.5 min, then returning to 98% CO_2_ in 0.8 min and re-equilibration till the end of the run; the column was held at the relevant temperature (35, 40, 45 or 55 °C) and the flow-rate was kept constant at 1.0 mL/min throughout the run. Prior to all gradient analyses, columns were equilibrated at 1 mL/min for 20 min (>10 column volumes) at initial gradient conditions. 4 × 1 μL injections of caffeine were then made, and the observed retention time stability and peak area variation under gradient conditions were used to confirm column equilibration. This was followed by 2 μL injections of each test mixture. For human urine analysis, the same chromatographic gradient was used on both the BEH 2-EP and the Torus Diol columns at 40 °C, using either 5% water in methanol or 20 mM ammonium formate in methanol as modifier; 1 μL aliquots of 1:3 urine:methanol extract were injected onto the column.

#### 2.3.5 Mass spectrometry for method development

MS analysis for method development was performed in ESI positive mode, with instrument configuration, source and analyser settings as given in Section 2.2.3, with the following modifications: extractor voltage 5 V and RF lens voltage 0.2 V. The flow from the PDA detector was mixed with 0.3 mL/min makeup solvent (0.1% v/v formic acid in methanol), supplied by a Waters 515 HPLC pump, before entering the MS source; the ABPR was held at 2000 psi (138 bar) for all analyses. When alkylamines were used as modifier additives, the UPC^2^ was used in stand-alone mode i.e. with UV–vis detection only.

#### 2.3.6 Data analysis

Retention time (t_R_), peak area, peak base width, peak asymmetry (b/a) and signal-to-noise (S/N) values for each analysis were obtained by processing the raw data in TargetLynx. Further statistical treatment was performed in R [28]. Venn diagrams were generated using the *VennDiagram* package [29]. Tukey-style boxplots (with no outliers shown) were generated using the default *graphics* package, with whiskers extending to (at a maximum) 1.5× the inter-quartile range (IQR); *n* values accompanying each boxplot or in the figure captions describe the number of data points included per condition.

## 3 Results and discussion

### 3.1 Screening of polar urinary metabolites for test compound identification

**Fig. 1:**
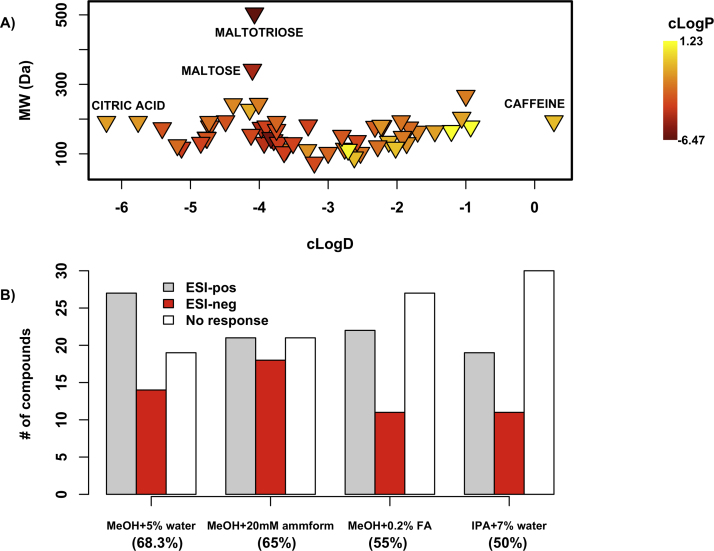
**A** shows the composition of the polar analyte library screened for test compound identification. A cLogP-based colorscale has been used in **A**, with yellow corresponding to higher cLogP values and dark red corresponding to the lower cLogP values; maltose and maltotriose had the highest molecular weights in the library, while caffeine and citric acid had the highest and lowest cLogD values respectively. **B** compares analyte hit-rate by co-solvent; the percentage of analytes eluting with acceptable peak descriptors in each co-solvent is listed in parentheses below the *x*-axis. (For interpretation of the references to colour in this figure legend, the reader is referred to the web version of this article.)

For the planned SFC method development study, it was necessary to identify a set of representative polar compounds that eluted with reasonable peak shapes and retention times in a variety of routinely-used chromatographic conditions. Accordingly, a library of 60 polar analytes of known biological significance or clinical relevance was screened using a BEH 2-EP column and an SFC gradient method with one of four different organic modifiers. The compound library (Fig. 1A, Table S1) included nucleobases and related compounds (e.g. uracil, adenosine, cytosine and cytidine), amino acids (e.g. methionine, tryptophan, lysine etc.,), organic acids (such as 2-aminobutyric acid, lactic acid) and sugars (such as glucose, galactose). The BEH 2-EP column was chosen for compound screening since it has been widely used for SFC separations of polar analytes. Similarly, the four selected modifiers have previously been used for polar compound analysis by SFC [26], [15].

Any analyte which eluted with an acceptable peak shape (as measured by b/a < 4), 2 ≤*k* ≤ 20 and a S/N ≥ 3 in either MS mode was considered to be a ‘hit’; Fig. 1B summarises the corresponding hit-rate for all tested modifiers. Compounds that were ‘hits’ in all modifiers were classified as ‘responders’, and subsequently formed the basis of chromatographic test mixtures for SFC method development. ‘Non-responders’ were those compounds for which peaks were not observed in the presence of any modifier, in any MS mode, and were accordingly left out of chromatographic test mixtures. Fig. S1 provides further details regarding the distribution of responders and non-responders in the various tested modifiers and MS modes.

### 3.2 Evaluation of chromatographic conditions

Having selected a suitable subset of polar analytes for test mixture preparation, a method development study was performed to evaluate chromatographic performance in a total of 264 unique combinations of column, modifier additive and column temperature. The resultant dataset was information-rich and complex, with a wide range of peak base widths and retention times exhibited by the twenty analytes across the tested conditions. In order to reduce this complex dataset to a more tractable form, it was necessary to identify criteria that would simplify evaluation and comparison of the separation quality. As high-throughput untargeted LC- or SFC-MS metabolic phenotyping studies require high peak capacities and resolution within a short analysis time, the quality of separation for each combination of column, modifier and temperature was evaluated based on three parameters: peak base width, sum of resolution and the normalised product of the resolution.

The peak base width may be related to peak capacity as follows:

(1) $$P_{c}=1+\frac{t_{g}}{\frac{1}{n}\sum_{1}^{n}w_{p}}$$

where *P_c_* is the peak capacity for a gradient of length *t_g_*, and *w*_p_ is the base width for each of *n* peaks used in the calculation [30]. Another common definition of peak capacity uses the sum of the resolution ($\Sigma {\text{R}}_{\text{s}}$) across the chromatogram:

(2) $$P_{c}=1+\underset{n}{\sum }R_{S}$$

where *R_S_* is the resolution between peaks, calculated here using Eq. (3):

(3) $$R_{S}=\frac{2\left(t_{r,1}-t_{r,2}\right)}{\left(w_{p,1}+w_{p,2}\right)}$$

where *w_p,n_* is the peak width for the nth peak, eluting at retention time *t_r,n_*. Both definitions for peak capacity have constraints on their use to describe gradient separations (e.g. Eq. (2) applies to situations where peak widths follow a similar pattern across the chromatogram [30]), but are sufficient for our purpose *viz*. comparison of separation quality under different chromatographic conditions.

Thus, based on Eqs. (1) and (2), the average peak base width and the average $\Sigma {\text{R}}_{\text{s}}$ may be used to compare the relative peak capacities of the evaluated chromatographic conditions. In the current analysis, we have chosen to compare peak base width and resolution data using the boxplot representation, and hence are comparing the median rather than the average parameters across conditions; however, where relevant, we have also provided mean values within the text.

One shortcoming of peak capacity as an estimate of chromatographic performance is that it does not take into account the distribution of peaks across the chromatographic window. While minimising peak base width and increasing resolution are crucial for improved separation in metabolic profiling studies, an even distribution of peaks across the retention time range ensures a more efficient use of chromatographic space. To evaluate peak distribution, Schoenmakers and others have used the normalised resolution product ($\underline{r}$ or NPR) [31]:

(4) $$\underline{r}=\underset{n}{\prod }(R_{S}/{\bar{R}}_{S})$$

where ${\bar{R}}_{S}$ is the mean resolution for *n* peaks. Thus for *n* peaks, each with the same $R_{S}$, the $\underline{r}$ would have a value of 1; separations with a more uneven distribution of peaks would have values $\underline{r}$ << 1. By considering the peak capacity together with the $\underline{r}$, a separation can be simultaneously optimised for greater analyte resolution and more even feature distribution.

In the following sections, boxplots comparing peak base widths across modifiers or columns are based on data for a subset of polar analytes which eluted with reasonable peak shapes and *k* values in all investigated conditions, *viz*. 2-aminobutyric acid, adenine, adenosine, creatinine, cytosine, hippuric acid, leucine, proline, uracil and xylitol. These analytes were selected in order to compare modifiers or columns with different *total* numbers of observed peaks i.e. in order to ensure that the comparison was based upon peaks observed in all evaluated conditions. On the other hand, the sum of resolution for each condition was necessarily calculated based on *all* observed peaks. Similarly, the $\underline{r}$ calculations used data from all observed peaks per condition. Note that the BEH Phenyl phase is excluded from all column performance evaluation plots shown below, due to the extremely poor peak shapes and separation observed when it was used for this set of analytes, regardless of the modifier additive or temperature used.

#### 3.2.1 Effect of modifier additives

**Fig. 2:**
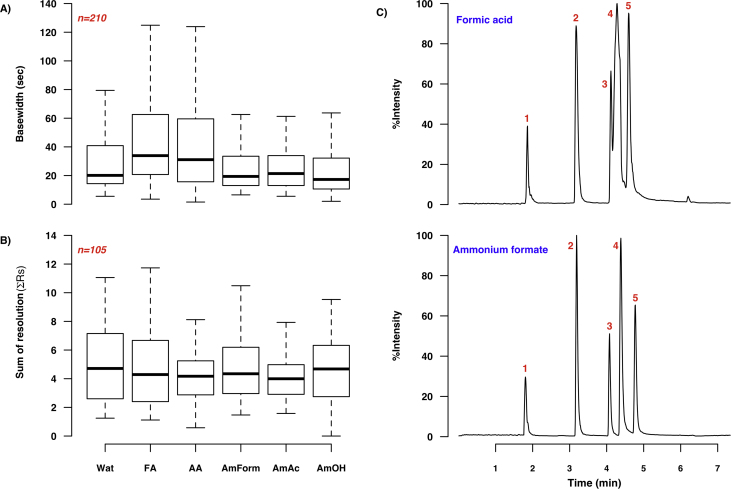

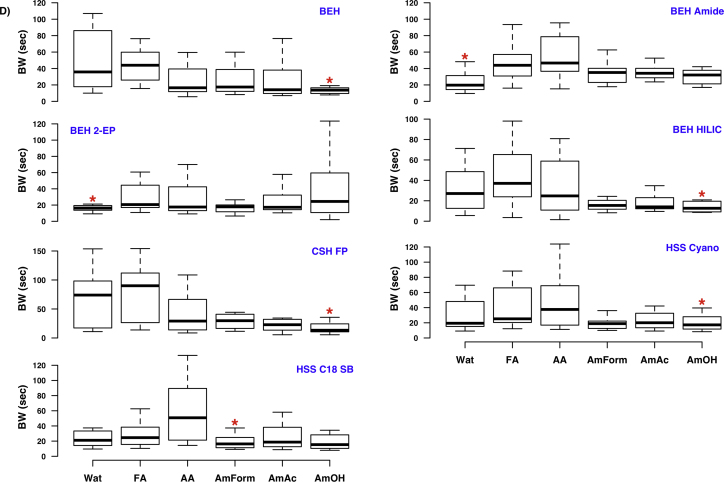
Effect of additive selection on chromatographic performance in SFC-MS. **A** shows the variation in peak base widths and **B** the differences in the sum of the resolutions (for all evaluated columns and temperatures) with co-solvent additive. **C** illustrates the improvement in separation observed when using 20 mM ammonium formate (AmForm) as a modifier additive rather than 0.5% v/v formic acid (FA). Both TIC chromatograms show separation of the nucleobase test mixture (**1**:caffeine, **2**:uracil, **3**:adenosine, **4**:cytosine and **5**:cytidine) on a Torus Diol column (3.00 i.d. × 100 mm, 1.7 μm d_P_) at 40 °C, using the 7.35 min gradient described in Section 2.3.4. **D** shows the effect of additive selection on peak base widths for all 7 columns in the initial test set, further expanding on the data shown in **A**; *n* = 30 for all plots and the red asterisks indicate the lowest median peak width value. (For interpretation of the references to colour in this figure legend, the reader is referred to the web version of this article.)

**Fig. 3:**
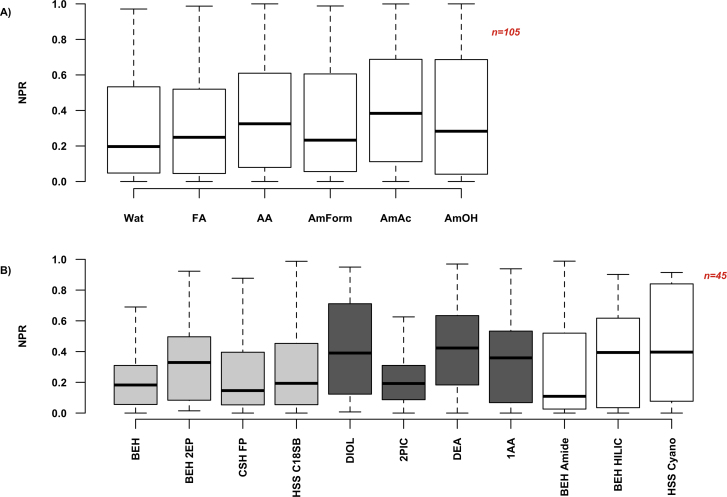
Normalised product of resolution ($\underline{r}$ or NPR) for SFC-MS data. **A** shows the variation in NPR across modifier additives (for all evaluated columns and temperatures), while **B** illustrates NPR variation across columns (for water, ammonium formate and formic acid-containing modifiers, at all evaluated temperatures); UPC^2^ columns are in light gray, Torus UPC^2^ columns are in dark gray and UPLC columns are in white.

A total of nine modifier additives were tested during method development; of these, water, formic acid, acetic acid and the ammonium salts were MS-compatible. The remaining three additives were alkylamines, and their use was consequently restricted to SFC-UV–vis only. The impact of additive selection upon peak base widths and resolution in SFC-MS mode is shown in Fig. 2: the three ammonium salts reduce the median peak base width considerably when compared to the acidic additives. This reduction is particularly pronounced for nucleobases and amines, in keeping with the basic nature of the ammonium salt additives. Thus the addition of ammonium hydroxide to the modifier resulted in a median peak base width of 17.3 s, as opposed to formic acid with a median width of 33.9 s (mean values are 26.5 and 46.6 s respectively). The use of ammonium formate and water also resulted in narrower peaks, with median widths of 19.4 (mean of 25.6 s) and 20.1 s (mean of 33.2 s) respectively. As illustrated by Fig. 2C and D, these general trends are evident in the performance of individual columns, and are even more pronounced for specific columns. However, the sum of resolutions ($\Sigma {\text{R}}_{\text{s}}$) was not significantly enhanced by any additive, ranging from a median value of 4.0 for ammonium acetate to 4.7 for water (Fig. 2B). In addition, peak distribution measured by $\underline{r}$ (Fig. 3A) followed a different trend, with the most even distribution seen for ammonium acetate (median $\underline{r}$ of 0.39, mean of 0.42), and the most uneven observed for ammonium formate and water, with median $\underline{r}$ values of 0.23 and 0.20 respectively. Different modifier additives may thus be chosen based on the desired outcome e.g. ammonium acetate for more even peak distribution, ammonium hydroxide for reduction in peak width, or ammonium formate for reduced peak widths across a wider range of analytes.

These results are, for the most part, in agreement with previous studies showing the utility of volatile ammonium salts for SFC-MS separation of polar molecules. Both ammonium formate and ammonium acetate increased the elution of highly polar pharmaceutically-relevant analytes from a cyano column [19], and both were found to improve chromatographic peak shapes for a range of doping agents separated on BEH and BEH 2-EP columns. The latter study also tested the variation in MS response with modifier additive and found that, in comparison to formic acid or ammonium hydroxide, both ammonium acetate and ammonium formate improved signal intensity for the majority of tested doping agents. Furthermore, the combination of ammonium formate with a small percentage (2% v/v) of water resulted in additional gains in signal intensity, and this combined mobile phase was subsequently used for the analysis of 110 doping agents in human urine by SFC-MS [32]. The current base width data supports the use of water as an additive to improve peak shapes for acids and sugars, while its use has previously been demonstrated to give sharper peaks for nucleobases on a variety of stationary phases, including diol, cyanopropyl and 2-EP [26]. In the current study, cytidine, the most strongly retained of the nucleobases tested, only eluted from the amide column when 5% water was used as an additive: this supports the findings of Taylor and other groups, and suggests that water could be used in combination with other additives to improve separation of the most polar analytes [20], [26]. Hamman et al. have demonstrated that 0.1% ammonium hydroxide in methanol as a modifier improves peak shape and decreases retention of basic drugs on both ethylpyridine and diol columns [33], and have also tested the stability of silica-supported chiral stationary phases in the presence of this modifier under SFC conditions. Their results suggest only minor stationary phase degradation occurs over 100,000 column volumes. Thus, the four additives with the greatest reduction in median peak base width (ammonium formate, acetate, hydroxide and water) should all be considered for the analysis of polar urinary metabolites by SFC-MS, with the final choice of additive (or additive combination) determined by the desired outcome, such as the targeted analysis of a specific compound class.

The three alkylamine additives were tested using UV–vis (PDA) detection only, due to concerns regarding ion suppression and MS source contamination. Their impact on chromatographic performance was evaluated based on the separation of the seven UV-active analytes present in the test mixtures *viz*. adenine, adenosine, creatinine, cytidine, cytosine, hippuric acid and uracil. Alkylamines have been used extensively as ion-pairing agents in LC, to increase retention and improve peak characteristics for small, basic analytes. Berger and Wilson showed that baseline resolution of drug mixtures (including a series of anti-depressants, anti-psychotics and stimulants) could be obtained by adding 0.5% v/v isopropylamine to a methanol-based SFC mobile phase [34], [35], [36], while De Klerck et al. have used isopropylamine in conjunction with TFA to increase enantioresolution in chiral SFC [37]. Similarly, Regalado et al. used 25 mM isobutylamine as an additive in methanol for chiral and achiral SFC separations of a variety of drugs and drug metabolites [38]. Thus, both isobutyl- and isopropylamine were included in our modifier screen, along with isopentylamine to determine if the size of the alkyl side-chain contributes to chromatographic performance in SFC.

**Fig. 4:**
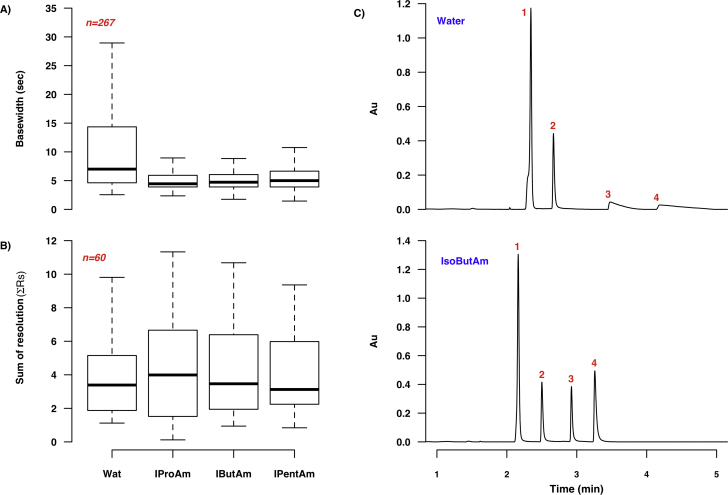

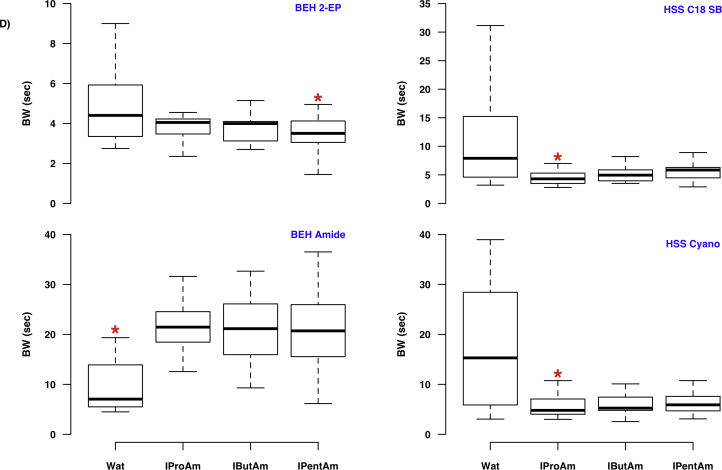
Effect of alkylamine additives on chromatographic performance in SFC (UV-vis data only). **A** shows the variation in peak base widths and **B** the differences in the sum of the resolutions (across all evaluated columns and temperatures); the *N* value of 267 in **A** reflects the fact that some analytes were present in multiple test mixtures. **C** illustrates the improvement in separation observed when using 0.5% v/v isobutylamine (IsoButAm) as a modifier additive rather than 5% water (Wat). Both chromatograms show separation of the nucleobase test mixture (**1**:uracil, **2**:adenosine, **3**:cytidine, **4**:cytosine) on an HSS Cyano column (3.00 i.d. × 100 mm, 1.8 μm d_P_) at 40 °C, using the 7.35 min gradient described in Section 2.3.4. **D** shows the effect of additive selection on peak base widths for 4 representative columns, further expanding on the data shown in **A;***n* = 39 for all plots and the red asterisks indicate the lowest median base width value. (For interpretation of the references to colour in this figure legend, the reader is referred to the web version of this article.)

The reduction in median peak base width obtained upon addition of alkylamines to the mobile phase is shown in Fig. 4. The UV–vis data showed consistently narrower peaks than the MS data, as is evident when comparing median peak widths for the methanol/5% water modifier in Figs. 2 A and 4 A. This difference between MS and UV data may indicate that further optimisation of MS settings and/or system volumes between column outlet and MS inlet is required. As shown in Fig. 4A, both the median peak base width and the peak base width range across conditions was significantly reduced in the presence of the alkylamine additives, when compared to results obtained with methanol/5% water as a modifier (the latter had a median peak base width of 7.0 s). The reduction in peak base width followed a clear trend based on the size of the alkyl side-chain, with isopropylamine showing the lowest median width (4.5 s) and isopentylamine showing the highest (5.0 s). The median $\Sigma {\text{R}}_{\text{s}}$ parameter decreased in the same direction, from 4.0 s for isopropylamine to 3.1 s for isopentylamine. Mean values for both peak base widths and $\Sigma {\text{R}}_{\text{s}}$ followed the same trends. These results are in keeping with the findings of Berger, Regalado and others, and suggest that alkylamine additives should be considered for SFC-UV–vis analysis of polar metabolites. Furthermore, while the evaluated alkylamines dramatically improve separation on all columns (except BEH Amide), smaller branched-chain alkyl groups appear to be preferable to larger ones. Amine additives are expected to decrease non-specific interactions between analyte and stationary phase by masking exposed silanols [39]. Thus, the observed size-dependent trend indicates that increasing the bulk of the akyl group reduces the interaction between amine and silanol groups, reducing the masking effect seen with smaller alkyl groups. As Fig. 4D illustrates, these general findings are also valid in the case of individual columns, with some exceptions: the use of isopentylamine as an additive resulted in the narrowest peaks for the BEH 2-EP column, while it was water rather than the alkylamines that provided the greatest decrease in peak widths for the BEH Amide column (7.1 s for water vs. 20.7 for isopentylamine).

#### 3.2.2 Effect of column chemistry

**Fig. 5:**
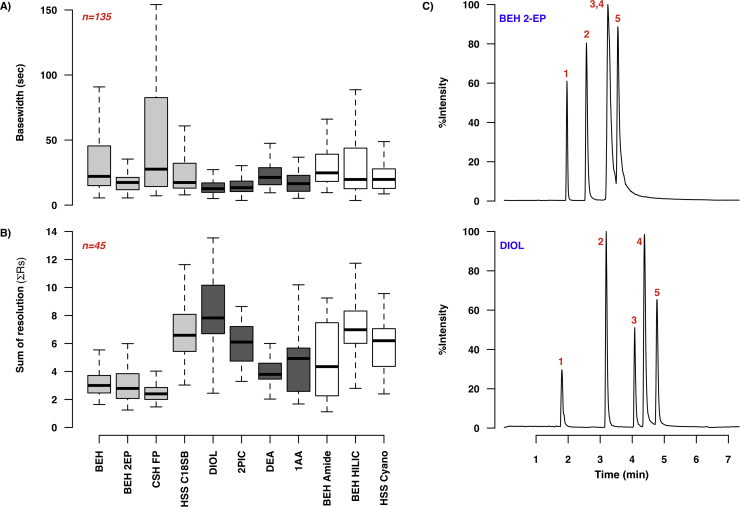

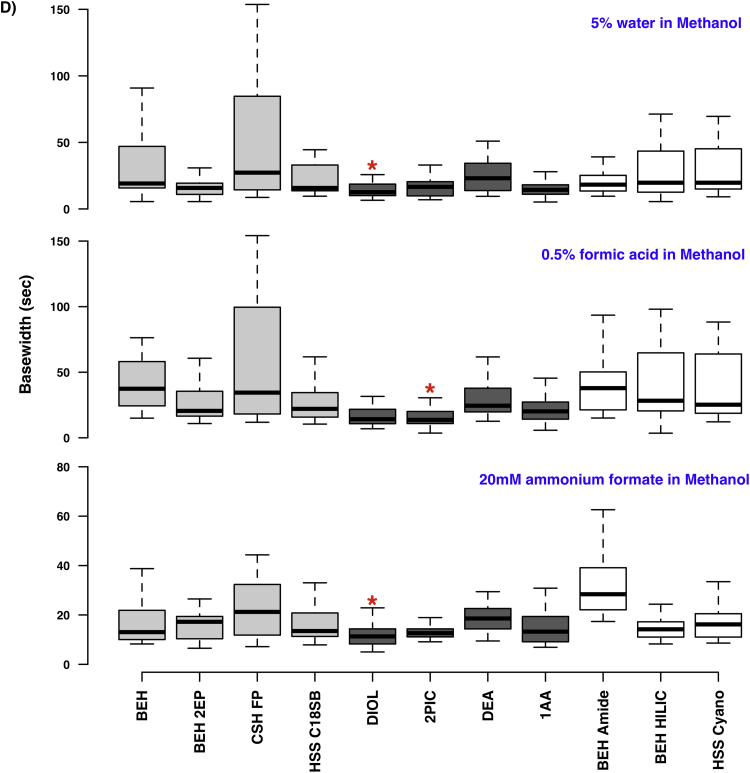
Effect of column selection on chromatographic performance in SFC-MS. **A** shows the variation in peak base widths and **B** the differences in the sum of resolutions (across water, ammonium formate and formic acid-containing modifiers and all evaluated temperatures) by column. UPC^2^ columns are in light gray, Torus UPC^2^ columns are in dark gray and UPLC columns are in white. **C** illustrates the improvement in separation observed when using a Torus Diol column rather than a BEH 2-EP column. Both TIC chromatograms show separation of the nucleobase test mixture (**1**:caffeine, **2**:uracil, **3**:adenosine, **4**:cytosine and **5**:cytidine) using 20 mM ammonium formate in methanol as a modifier at 40 °C, using the 7.35 min gradient described in Section 2.3.4. **D** shows the effect of column selection on peak base widths for 3 representative co-solvents, further expanding on the data shown in A; *n* = 45 for all plots and the red asterisks indicate the lowest median base width value. (For interpretation of the references to colour in this figure legend, the reader is referred to the web version of this article.)

A total of twelve stationary phases were evaluated during method development. Of these, eight were columns specifically marketed for SFC use, while the remaining four were columns for use with RPLC or HILIC separations on UHPLC systems. The tested SFC columns included both the recently introduced ACQUITY UPC^2^ Torus columns, designed for improved robustness and stability in mixed CO_2_-methanol mobile phases [7], [40], and the older ACQUITY UPC^2^ range. The effect of column selection on peak widths and on the sum of resolution across a range of modifiers and temperatures in summarised in Fig. 5, while Fig. 3B shows the differences in $\underline{r}$ due to column type. From Fig. 5A it is evident that the greatest reduction in peak base width was obtained by use of the Torus range of columns (in dark grey), and that the peaks on this column range were generally narrow. The Diol column had the lowest median peak width of 12.6 s (mean of 14.1 s), though the 2-PIC column had a very similar median width (13.4 s) (mean of 15.3 s). The only non-Torus columns to show comparable median peak width reduction and peak width range were the HSS C18 SB and BEH 2EP columns (median widths of 17.4 for both); however, as is evident in Fig. 5C, peak resolution on the Diol column was markedly better than on the BEH 2-EP column. The UPLC columns (in white) showed slightly higher median peak widths, but the variation in values across conditions was much higher than for the Torus UPC^2^ columns. The largest variation in peak width was observed for the CSH FP column, which also showed a relatively uneven distribution of peaks (Fig. 3B). The trend in peak width values was also seen for every compound class, with both Diol and 2-PIC columns showing significant reductions in peak width for all classes of analyte. Indeed, all Torus columns showed marked reductions in both median peak base widths and the base width variation (range) for all tested analyte classes. When column performance was compared in individual co-solvents (Fig. 5D) it became apparent that the overall trends observed in Fig. 5A and B were also applicable in specific cases: the Diol column had the lowest median peak widths in the presence of water and ammonium formate (12.6 and 11.3 s respectively), while the 2-PIC column had the lowest median width in formic acid (13.7 s). Note that Fig. 5D also demonstrates the beneficial effect of using ammonium formate as an additive for this set of analytes, with narrower peak widths observed across all column types in the presence of this additive.

Of the Torus columns, the Diol column also had the highest median $\Sigma {\text{R}}_{\text{s}}$ value (7.8) in keeping with the peak base width reduction, and a relatively high median $\underline{r}$ of 0.39 (Fig. 3B). In general, the more even peak distributions were observed for the Torus columns (with the exception of the 2-PIC), the BEH HILIC and HSS Cyano columns, while the BEH Amide and CSH FP had the most uneven distributions. Thus the Torus columns, and in particular the Diol column, offer improved resolution due to narrower peak base widths for all classes of polar analyte, and a relatively even distribution of peaks across the retention time range.

#### 3.2.3 Effect of temperature

The temperature range initially evaluated (35–45 °C) was selected because it encompasses the common operational temperature range for untargeted LC–MS-based urine metabolomics [41], and is also well below the maximum recommended temperature for the majority of screened stationary phases (60 °C for the UPC^2^ columns and 90 °C for the UPLC columns). The ABPR pressure setting used (2000 psi/138 bar) ensured that on-column phase separation of the methanol-CO_2_ mixture was not expected to occur in the evaluated temperature range [42], and the relatively high proportion of modifier meant that the mobile phase density remained predominantly liquid-like during the gradient run. Thus, increasing the temperature can be expected to impact retention and resolution in a similar manner as in LC.

**Fig. 6:**
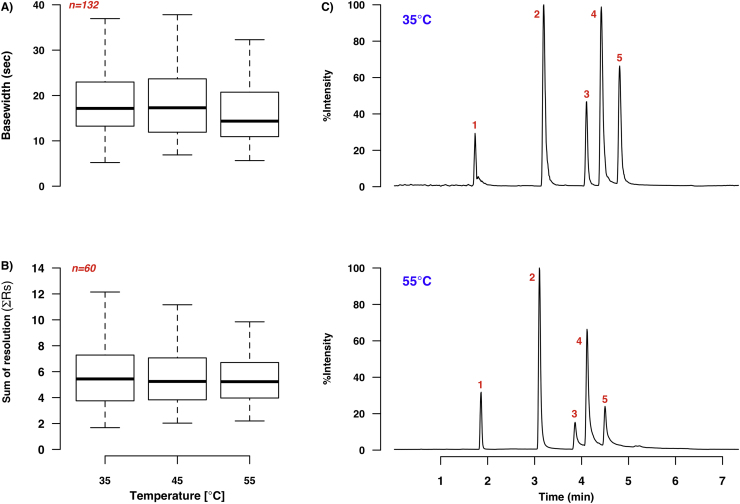

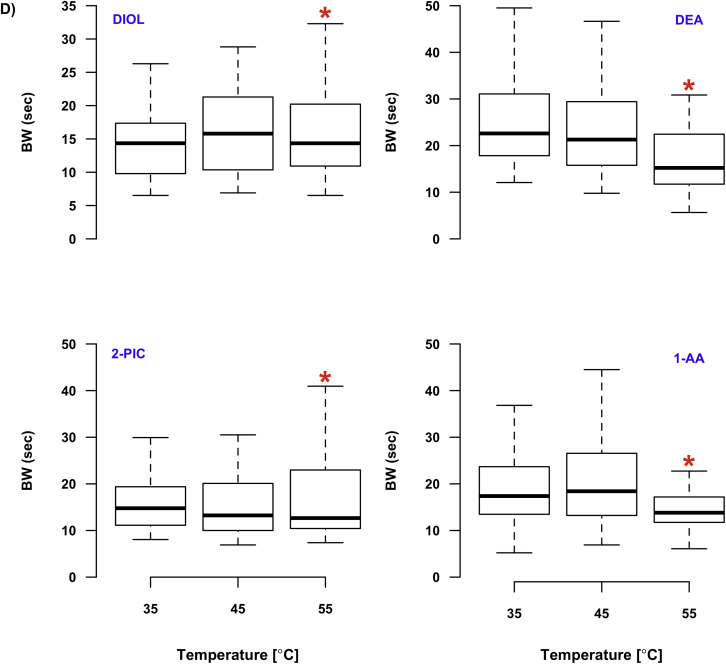
Effect of temperature on chromatographic performance in SFC-MS. **A** shows the variation in peak base widths and **B** the differences in the sum of resolutions (across water, ammonium formate and formic acid-containing modifiers and all Torus columns) by temperature. **C** illustrates the change in peak width and resolution observed when using a Torus DIOL column at 55 °C rather than at 35 °C. Both TIC chromatograms show separation of the nucleobase test mixture (**1**:caffeine, **2**:uracil, **3**:adenosine, **4**:cytosine and **5**:cytidine) using 20 mM ammonium formate in methanol as a modifier at the relevant temperature, using the 7.35 min gradient described in Section 2.3.4. **D** shows the effect of temperature selection on peak base widths for the Torus columns, further expanding on the data shown in **A**; *n* = 33 for all plots and the red asterisks indicate the lowest median base width value. (For interpretation of the references to colour in this figure legend, the reader is referred to the web version of this article.)

All three temperatures initially studied showed similar median peak base width and $\Sigma {\text{R}}_{\text{s}}$ values, with no definite trend observable for any particular class of compound (Fig. S2A and B). The results suggested that increasing temperature over this range conferred no significant advantage in terms of chromatographic performance; no consistent trends were discernable even when the temperature behaviour of individual columns or co-solvents (Fig. S2C and D respectively) was inspected. Accordingly, the temperature range was extended by testing one additional temperature, 55 °C, which is still below the maximum temperature recommended for the UPC^2^ columns. Only the Torus columns were screened at this higher temperature; the results are illustrated in Fig. 6. While changing temperature from 35 to 45 °C did not significantly alter the median peak widths or total resolution, increasing temperature to 55 °C resulted in a 3 s drop in median base width (from 17.2 s at 35 °C to 14.3 s at 55 °C). However, median $\Sigma {\text{R}}_{\text{s}}$ values did not improve over the same temperature range, perhaps since most analytes eluted earlier at 55 °C, so reducing resolution (Fig. 6C).

The reduction in median peak width at 55 °C was also observed for each column evaluated at this temperature (Fig. 6D). Together, these results suggest that peak capacity may be improved by increasing column temperature to 55 °C, but that such an increase should be carefully weighed against the possible reduction in absolute resolution that accompanies the temperature change. In addition, since the highest recommended temperature for the Torus columns is 60 °C, it may be wise to select a lower operating temperature to ensure column stability over the course of high-throughput metabolomics experiments.

#### 3.2.4 Analytical repeatability^1^

Within-day analytical precision was measured by monitoring the retention time and peak area variation of caffeine (present in each test mixture as an internal standard). The caffeine peak eluted relatively early in all conditions, when the mobile phase consisted predominantly of CO_2_ (rather than the organic modifier). This meant that the caffeine peak was also more susceptible to spray-pulsing or spray stability phenomena seen at the MS inlet in high percentages of CO_2,_ and thus functioned as a sensitive indicator of such problems. As shown in Figs. S3 and S4, while the variation in retention time (as measured by% RSD) was generally quite small, at <2% in all conditions, the area of the caffeine peak varied significantly both across modifiers and column types. Ammonium hydroxide had the highest median caffeine peak area variation of all the MS-compatible additives (16.3% vs. 10.0% for ammonium formate), meaning that the improvements in resolution obtained with this additive were accompanied by increased analytical variation. Surprisingly, both Diol and 2-PIC columns, with narrow peaks and higher sum of resolution, showed the highest variation in median caffeine peak area (16.9 and 13.9% respectively). Such high variation compares unfavourably with the BEH 2-EP column, which has the lowest peak area RSD of 2.1%. Given the low variability in retention time values across columns, the large variations in caffeine peak area were investigated further, and found to be indicative of spray pulsing into the ESI source. As such, this variation can be addressed by increasing the percentage of organic modifier in the mobile phase at the start of the gradient. Preliminary tests show that increasing the modifier content from 2 to 10% at the start of the gradient significantly reduces the observed variation in the caffeine peak area, and has the added advantage of reducing the gradient length without affecting overall separation. Since all of the tested polar analytes require the presence at least 14% modifier in the mobile phase for elution, this increase in modifier content is unlikely to significantly reduce the efficacy of the method, even for early-eluting analytes.

#### 3.2.5 Analysis of human urine extract and polar standards

The method development study described herein indicates that of the tested Waters columns, the Torus range should be the first choice for the analysis of any class of polar metabolite; similarly, the use of either ammonium salts or water as modifier additives should be considered when analysing such compounds using SFC. In order to confirm these findings, human urine was analysed using the 7.35 min gradient method on the Torus Diol column in the presence of either 5% water or 20 mM ammonium formate in methanol as a modifier. The results were compared to the analysis of the same urine sample on the BEH 2-EP column using the same set of modifiers, since this column is widely used for polar compound analysis by SFC.

**Fig. 7:**
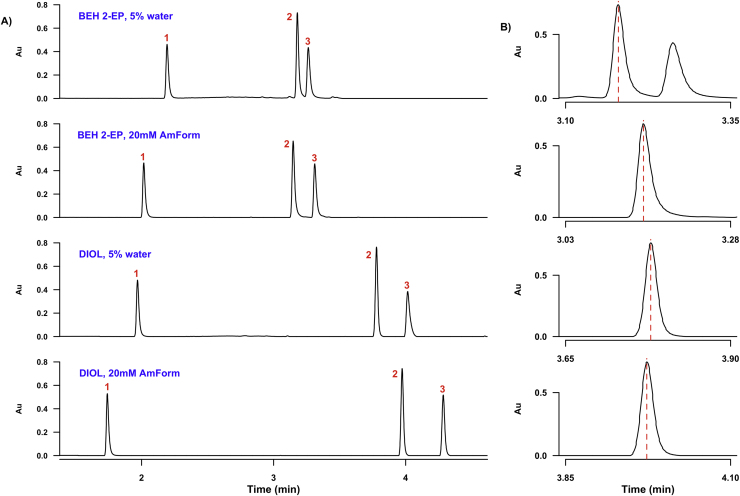
Results of method development study for SFC separation of polar urinary metabolites. **A** illustrates the effect of column and modifier selection on chromatographic separation for polar analyte standards spiked into human urine (**1**:caffeine, **2**:cytosine and **3**:uridine); in B, the cytosine peak from each separation shown in **A** is magnified, to show the improvement in peak symmetry upon replacement of the BEH 2-EP column with the Torus Diol column.

The utility of the findings from the method development study are evident in Fig. 7, which shows the pronounced improvement in peak capacity and overall resolution upon selection of a suitable modifier additive and column. In Fig. 7A, the use of ammonium formate rather than water as modifier additive resulted in baseline separation of uridine and cytosine on the BEH 2-EP column and improved resolution on the Diol column; however, when the BEH 2-EP column was replaced by the Diol column, both peak shape (Fig. 7B) and resolution improved, regardless of which additive is used. Other Torus columns such as the 2-PIC can be used instead of the Diol without a significant loss of resolution or peak symmetry, and the combination of ammonium formate and water as modifier additives might improve separation more than the use of either one by itself. Overall, however, the Torus Diol column used with a methanol/ammonium formate modifier is a promising starting point for untargeted SFC analysis of medium polarity metabolites in urine. This was further confirmed by the elution of 45 out of the 60 compounds in the initial library using this combination of column and modifier at 40 °C (Table S1), i.e. these conditions resulted in a 75% hit-rate for the polar metabolite library of interest, with the majority of analytes exhibiting excellent peak shape with little tailing. The majority of analytes which were not detected under these conditions were organic acids, such as lactic acid, and amino acids such, as histidine: in order to detect these, additional optimisation of MS source or solvent conditions, or the use of MRM analyses, may be required. Interestingly, adding both water and ammonium formate to the co-solvent resulted in a lower hit-rate of 63%, all other conditions (column, temperature etc.) remaining the same; this suggests that simply combining modifier additives is not sufficient to improve performance, but must be accompanied by an optimisation procedure for each set of analytes.

## 4 Conclusions

The diversity of the molecular species involved in metabolic pathways is such that nearly any analytical platform is a viable tool for metabolic phenotyping. For UHPLC–MS based metabolic profiling, RPLC is the preferred mode for the separation of many non-polar metabolites, such as lipids, while HILIC is often used to analyse more hydrophilic compounds; the polarity range covered by these chromatographic methods is substantial. Yet profiling certain compound classes remains challenging, either because they are poorly retained under RP conditions due to their high polarity, or because a robust HILIC method for the same analytes requires prolonged re-equilibration times between analyses [23]. In addition, both RP and HILIC methods are dependent on an uninterrupted supply of high-purity organic solvents such as methanol and acetonitrile, which (in a high-throughput environment) can impose significant operational costs associated with solvent purchasing and disposal. SFC thus offers an alternative to solvent-hungry LC–MS-based metabolic profiling, while simultaneously increasing the range of analysable metabolites due to its orthogonality to RPLC under the appropriate conditions.

The results presented here demonstrate that SFC can indeed be used to successfully analyse the majority of polar urinary metabolites of interest in the cLogP range from 2 to −7. Of the twelve columns evaluated, the Torus columns were clearly preferable for such applications, with the Diol column in particular showing higher peak capacities (lower peak base widths and higher $\Sigma {\text{R}}_{\text{s}}$ values) for all analyte classes, and a somewhat more even distribution of peaks (higher median $\underline{r}$ values overall). In addition, the Torus range has been designed to avoid the pronounced and continuous retention time shifts observed in SFC using conventional phases with methanolic modifiers (which have recently been attributed to the formation of methyl silyl ethers under mixed CO_2_-methanolic conditions); thus, these columns are most likely suited for applications with prolonged analysis times [40]. Of the six MS-compatible additives studied, the ammonium salts generally resulted in lower base widths and higher overall resolution than the other additives; however, the increased analytical variation seen for ammonium hydroxide should be considered when selecting it as an additive. Alkylamine additives such as isopropylamine should also be considered for UV-active analytes, as these produced marked reductions in peak widths. However, the significant increase in peak capacities when using UV–vis detection *vs.* MS detection suggests further optimisation of MS settings and/or the interface between SFC and MS is required. Finally, while the use of higher temperatures can reduce peak widths for the majority of columns, the choice of temperature must also be based on stability considerations for high-throughput experiments.

## Footnotes

1 ‘Repeatability’ is used here rather than ‘reproducibility’ following NIST recommendations for use of these terms [43].
