# Supplementary material for: Analysis of polar urinary metabolites for metabolic phenotyping using supercritical fluid chromatography and mass spectrometry
Source: J Chromatogr A. 2016 Jun 3;1449:141–55. doi: 10.1016/j.chroma.2016.04.040 (PMC4927693; doi:10.1016/j.chroma.2016.04.040)
Supplement: Supplementary file 1 [file mmc1.doc]

**Supplementary Material**

**Tables**

**Table S1** Polar compound library screened by SFC-MS; those in italics were subsequently included in test mixtures for method development. The retention time data shown here was collected on a Waters Torus DIOL column (3.00 i.d. x 100 mm, 1.7 μm d_P_) at 40°C, using the 7.35 min gradient described in section 2.3.4, and one of two different organic modifiers: 20 mM ammonium formate in methanol and 10 mM ammonium formate in methanol with 2.5% v/v water. The observed retention time of toluene is included as an approximate indication of t_0_.

| **Compound** | **MW (g/mol)** | **cLogP (ChemAxon)** | **cLogD**  **(ACD/**  **Percepta Labs, pH 5.5)** | **Stock solvent** | **20 mM ammonium formate in methanol** | | **10 mM ammonium formate + 2.5% (v/v) water in methanol** | |
| --- | --- | --- | --- | --- | --- | --- | --- | --- |
|  |  |  |  |  | **Ret. Time (RT, min)** | **Ret. Time Std. Dev.** | **Ret. Time (RT, min)** | **Ret. Time Std. Dev.** |
| **Toluene** | **92.14** |  |  | **Methanol** | **0.23** | **0.00** | **0.23** | **0.00** |
| *2-aminobutyric acid* | *103.12* | *-2.32* | *-2.53* | *Water* | 4.55 | 0.01 | 4.26 | 0.01 |
| **2,4-diaminobutyric acid** | **118.13** | **-4.03** | **-5.13** | **Water** | **3.69** | **0.01** | **NA** | **NA** |
| 2'-deoxycytidine | 227.22 | -0.92 | -4.14 | Methanol | NA | NA | NA | NA |
| 3-phenyllactate | 166.17 | 1.18 | -1.21 | Methanol | 3.90 | 0.03 | 3.64 | 0.01 |
| *Adenine* | *135.13* | *-0.66* | *-2.12* | *Water* | *3.55* | *0.01* | *3.40* | *0.01* |
| *Adenosine* | *267.24* | *-2.09* | *-1* | *Water* | *3.91* | *0.01* | *3.78* | *0.01* |
| α-D-fucose | 164.16 | -1.89 | -1.7 | Methanol | 5.19 | 0.02 | 3.66 | 0.01 |
| α-galactouronic acid | 194.14 | -3.25 | -4.49 | Water | 3.15 | 0.01 | 3.14 | 0.02 |
| Arabitol | 152.15 | -3.10 | -2.8 | Water | 3.91 | 0.01 | 3.72 | 0.01 |
| *Arginine* | *174.20* | *-3.16* | *-5.41* | *Water* | *NA* | *NA* | *NA* | *NA* |
| Asparagine | 132.12 | -4.29 | -3.93 | Water | 6.20 | 0.01 | 5.53 | 0.02 |
| β-amino isobutyric acid | 103.12 | -2.41 | -3 | Water | 4.61 | 0.02 | 4.33 | 0.01 |
| *Caffeine* | *194.19* | *-0.55* | *0.28* | *Methanol* | *1.68* | *0.01* | *1.72* | *0.00* |
| *Choline chloride* | *139.62* | *-4.66* | *-3.79* | *Methanol* | *4.24* | *0.03* | *NA* | *NA* |
| Citric acid | 192.12 | -1.32 | -6.22 | Water | NA | NA | NA | NA |
| Citrulline | 175.19 | -4.65 | -3.97 | Water | NA | NA | 5.86 | 0.01 |
| *Creatinine* | *113.12* | *-2.28* | *-3.61* | *Methanol* | *3.53* | *0.01* | *3.40* | *0.01* |
| *Cytidine* | *243.22* | *-1.82* | *-4.38* | *Methanol* | *4.65* | *0.01* | *4.40* | *0.01* |
| *Cytosine* | *111.10* | *-1.56* | *-3.3* | *Methanol* | *4.27* | *0.01* | *4.04* | *0.01* |
| Dulcitol | 182.17 | -3.73 | -3.29 | Water | 4.31 | 0.01 | 4.13 | 0.01 |
| Galactosamine | 179.17 | -3.68 | -3.9 | Water | 5.29 | 0.04 | 5.09 | 0.02 |
| Galactose | 180.16 | -3.57 | -2.21 | Water | 4.30 | 0.02 | 4.13 | 0.01 |
| Gluconic acid lactone | 178.14 | -2.75 | -2.32 | Methanol | NA | NA | NA | NA |
| Glucosamine | 179.17 | -3.04 | -4.72 | Water | 5.26 | 0.02 | 5.03 | 0.01 |
| Glucose | 180.16 | -3.57 | -2.21 | Methanol | 4.36 | 0.02 | 4.20 | 0.01 |
| Glucurono-3,6-lactone | 176.12 | -2.58 | -1.82 | Methanol | 3.45 | 0.01 | 3.37 | 0.01 |
| Glutamic acid | 147.13 | -3.24 | -4.77 | Water | NA | NA | NA | NA |
| *Glutamine* | *146.14* | *-5.83* | *-3.83* | *Water* | *5.66* | *0.03* | *NA* | *NA* |
| Glycine | 75.07 | -3.41 | -3.2 | Water | 5.38 | 0.02 | 4.94 | 0.01 |
| Glycylglycine | 132.12 | -3.94 | -3.72 | Methanol | NA | NA | NA | NA |
| *Hippuric acid* | *179.17* | *1.23* | *-0.93* | *Methanol* | *4.19* | *0.01* | *3.99* | *0.00* |
| Histidine | 155.15 | -3.62 | -4.12 | Water | NA | NA | NA | NA |
| Isocitric acid | 192.12 | -1.45 | -5.76 | Water | NA | NA | NA | NA |
| L-serine | 105.09 | -3.89 | -3.64 | Water | 5.6 | 0.06 | 5.12 | 0.01 |
| Lactic acid | 90.08 | -0.47 | -2.62 | Methanol | NA | NA | NA | NA |
| *Leucine* | *131.17* | *-1.59* | *-1.86* | *Water* | *4.22* | *0.00* | *3.98* | *0.00* |
| Lysine | 146.19 | -3.21 | -4.76 | Water | NA | NA | NA | NA |
| *Maltose* | *342.30* | *-4.70* | *-4.1* | *Methanol* | *5.48* | *0.01* | *5.13* | *0.02* |
| Maltotriose | 504.44 | -6.47 | -4.07 | Methanol | NA | NA | NA | NA |
| Mannitol | 182.17 | -3.73 | -3.29 | Water | 4.28 | 0.01 | NA | NA |
| Mannose | 180.16 | -3.57 | -2.21 | Methanol | 4.22 | 0.01 | NA | NA |
| Meso-erythritol | 122.12 | -2.47 | -2.28 | Methanol | 3.36 | 0.01 | 3.31 | 0.00 |
| Methionine | 149.21 | -2.19 | -1.92 | Methanol | 4.65 | 0.01 | 4.33 | 0.01 |
| Methyl-α-D-glucopyranoside | 194.18 | -2.29 | -1.94 | Methanol | 3.52 | 0.01 | 3.43 | 0.00 |
| 1-methyl histidine | 169.18 | -3.40 | -3.75 | Water | 5.99 | 0.10 | 5.70 | 0.16 |
| Ornithine | 132.16 | -3.66 | -4.85 | Water | NA | NA | NA | NA |
| *Phenylalanine* | *165.19* | *-1.18* | *-1.45* | *Water* | *4.55* | *0.01* | *4.26* | *0.01* |
| *Proline* | *115.13* | *-2.57* | *-2.76* | *Methanol* | *4.44* | *0.02* | *4.14* | *0.01* |
| Quinic acid | 192.17 | -2.70 | -3.75 | Methanol | NA | NA | 5.41 | 0.06 |
| Saccharic acid 4-lactone | 192.12 | -2.42 | -4.73 | Water | NA | NA | NA | NA |
| Sorbitol | 182.17 | -3.73 | -3.29 | Methanol | 4.21 | 0.02 | 4.03 | 0.01 |
| Succinic acid | 118.09 | -0.40 | -2.02 | Methanol | 3.97 | 0.01 | 3.55 | 0.01 |
| *Taurine* | *125.15* | *-2.61* | *-5.19* | *Water* | *5.00* | *0.02* | *NA* | *NA* |
| Trans-4-hydroxy proline | 131.13 | -3.72 | -3.51 | Water | 4.81 | 0.01 | 4.49 | 0.01 |
| *Trigonelline* | *137.14* | *-3.53* | *-2.58* | *Methanol* | *4.68* | *0.03* | *4.29* | *0.01* |
| *Tryptophan* | *204.23* | *-1.09* | *-1.06* | *Water* | *5.69* | *0.02* | *5.17* | *0.01* |
| Tyrosine | 181.19 | -1.49 | -2.23 | Water | 5.19 | 0.01 | 4.84 | 0.00 |
| *Uracil* | *112.09* | *0.72* | *-2.71* | *Water* | *3.09* | *0.01* | *3.07* | *0.00* |
| Uridine | 244.20 | -2.05 | -4.01 | Water | 3.92 | 0.01 | 3.77 | 0.01 |
| *Xylitol* | *152.15* | *-3.10* | *-2.8* | *Methanol* | *3.88* | *0.01* | *3.66* | *0.00* |

**Table S2** Polar analyte test mixtures for chromatographic method development

| **Mixture** | **Components*** | **Concentration (mM)** |
| --- | --- | --- |
| **1** | 2-amino butyric acid  Hippuric acid  Maltose  Xylitol | 1  1  5  5 |
| **2** | Adenosine  Cytidine  Cytosine  Uracil | 2.5  2.5  0.5  1 |
| **3** | Choline  Creatinine  Proline  Taurine  Trigonelline | 2.5  2.5  0.5  5  5 |
| **4** | Creatinine  Cytidine  Glutamine  Hippuric acid  Leucine  Phenylalanine  Tryptophan | 1  1  1  1  1  1  1 |
| **5** | Adenine  Adenosine  Arginine  Hippuric acid  Phenylalanine  Taurine  Tryptophan  Uracil | 1  1  1  1  1  1  1  1 |

* All mixtures contained caffeine at 0.025 mM as an internal standard

**Supplementary Figures**

**Figure S1** Venn diagrams showing distribution of ‘responders’ (in ‘ESI-pos’ and ‘ESI-neg’ modes) and ‘non-responders’ (‘No response’) by co-solvent; analytes listed next to each figure are those which are common to all co-solvents (corresponding to the overlap region at the centre of the quadruple Venn diagram).

**
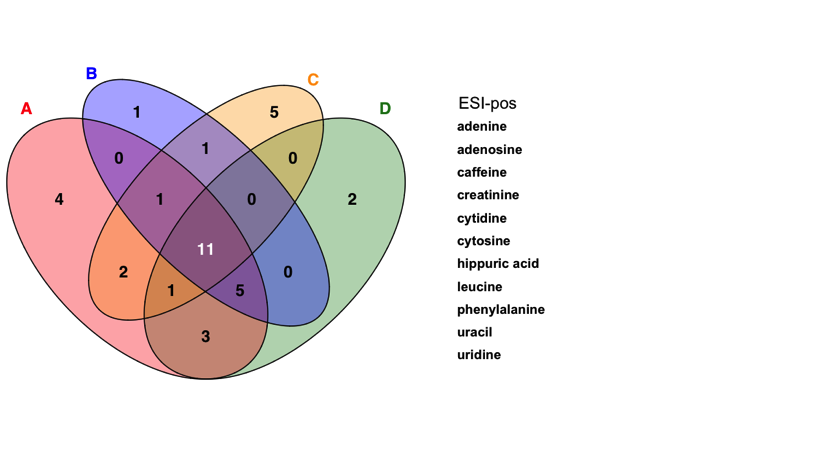
**

**
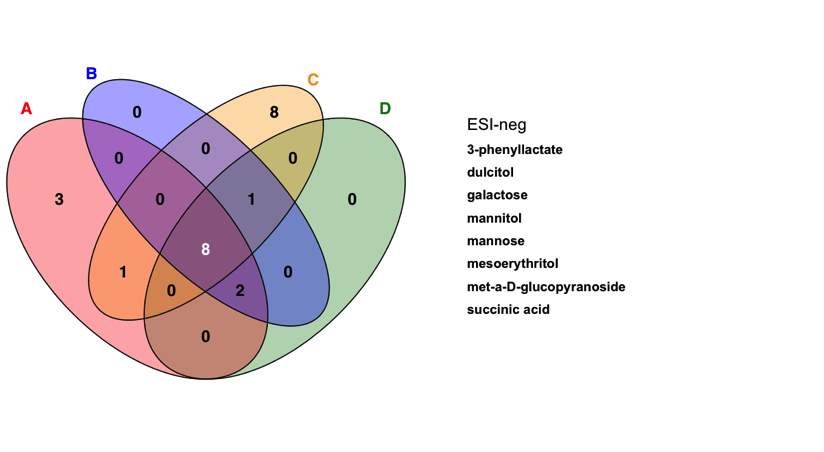
**

**
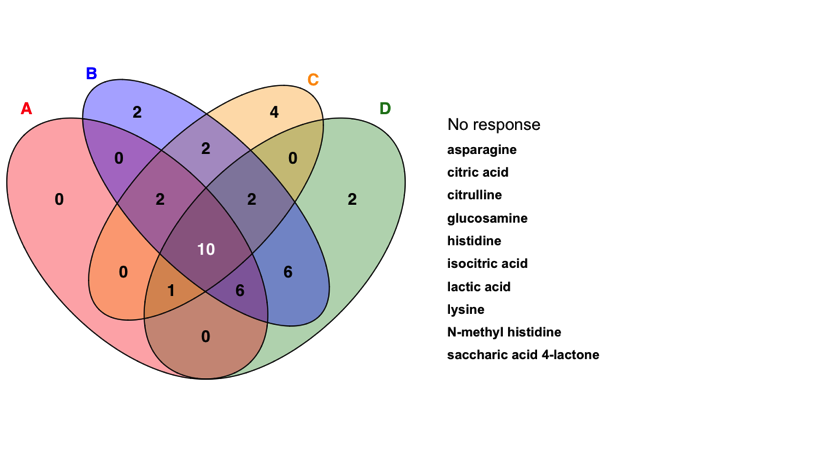
**

**Figure S2** SFC-MS peak basewidth (**A**) and
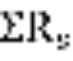
(**B**) distribution by temperature, across all columns and 3 modifier additives (water, formic acid, ammonium formate); **C** shows basewidth distribution by temperature for 4 representative columns (in 3 co-solvents, n= 30); **D** shows basewidth distribution by temperature for three selected co-solvents (on all columns, n = 110).

**Figure S3** Effect of co-solvent on variation in caffeine retention time (t_r_, **A**) and peak area (**B**) during gradient SFC-MS analysis. Data is shown for all evaluated columns and temperatures.

**Figure S4** Effect of column selection on variation in caffeine retention time (t_r_, **A**) and peak area (**B**) during gradient SFC-MS analysis. Data is shown for 3 co-solvents and all evaluated temperatures; UPC^2^ columns in are in light gray, Torus UPC^2^ columns in dark gray and UPLC columns in white.
